# Diabetes Prevention: Vitamin D Supplementation May Not Provide Any Protection If There Is No Evidence of Deficiency!

**DOI:** 10.3390/nu11112651

**Published:** 2019-11-04

**Authors:** Uwe Gröber, Michael F. Holick

**Affiliations:** 1 Academy for Micronutrient Medicine, Zweigertstr, 55, 45130 Essen, Germany; 2 Section of Endocrinology, Nutrition and Diabetes, Department of Medicine, Boston University Medical Center, Boston, MA 02118, USA; mfholick@bu.edu

**Keywords:** vitamin D, type 2 diabetes, 25-hydroxyvitamin D, prediabetes, glucose metabolism, insulin resistance

## Abstract

The results of epidemiological and several interventional studies suggest an association between vitamin D deficiency and an increased risk of developing insulin resistance or type 2 diabetes. Various studies have indicated that a lack of vitamin D must be regarded as a pathogenic factor for type 2 diabetes and the metabolic syndrome, since a vitamin D deficiency (25(OH)D < 20 ng/mL) increases insulin resistance and reduces insulin secretion from beta cells in the pancreas. A recent study by Pittas et al. did not show a clear preventive effect of vitamin D supplementation with respect to the risk of developing type 2 diabetes. In terms of this study, it must be remembered that more than 70% of the participants in both the vitamin D supplement group and the placebo group did not have a vitamin D deficiency. In medical and pharmaceutical practice, more attention should be paid to vitamin D deficiency than has previously been accorded. Vitamin D status can be assessed objectively when necessary by laboratory testing of the serum 25(OH)D levels. Type 2 diabetes patients benefit from improving their vitamin D status with respect to their glucose metabolism and decreased mortality risk. Patients with insulin resistance who are vitamin D deficient should be treated with an appropriate amount of vitamin D to achieve circulating levels of 25(OH)D of 40–60 ng/mL.

## 1. Introduction

According to WHO data, the prevalence of type 2 diabetes almost doubled worldwide between 1980 and 2014, with an estimated 422 million people suffering from diabetes mellitus in 2014 [1,2]. Obesity, type 2 diabetes, and vitamin D deficiency are pandemic diseases of our time, affecting millions of people throughout the world. The results of the latest epidemiological studies, interventional studies, and meta-analyses suggest an association between vitamin D deficiency and an increased risk of developing insulin resistance or type 2 diabetes. There have also been contradictory results from previous studies that do not focus on vitamin D deficient states. In this commentary, we reflect on how these findings can be explained [3,4,5,6,7].

## 2. Interventional Studies Have Yielded Contradictory Results

In a multicenter, randomized, placebo-controlled clinical trial published in the “New England Journal of Medicine”, the research group of Pittas et al. [8] from Tufts Medical Center in Boston wanted to clarify whether vitamin D_3_ supplementation could reduce the risk of disease in people with a high risk of diabetes. A total of 2422 patients, who each met at least two of the three diagnostic criteria for prediabetes (fasting glucose levels of 100–125 mg/dL, glucose levels of 140–199 mg/dL following a two hour oral glucose tolerance test, and HbA1c 5.7%–6.4%), took a daily dose of either 4000 IU vitamin D_3_ or a placebo for a period of 24 months.

It is worth noting that vitamin D deficiency (25(OH)D < 20 ng/mL) as defined by the Endocrine Society [9] was not one of the inclusion criteria of this interventional study. Even though the baseline 25(OH)D levels were measured, the patients were randomized into two groups irrespective of their initial vitamin D status. Only 276 (22.8%) of the 1211 patients in the active treatment group and 249 (20.6%) of the 1211 patients in the placebo group were vitamin D deficient (25(OH)D < 20 ng/mL) at the start of the study [9]. Thus, 77.2% of the patients in the active treatment group and 79.4% in the placebo group were not vitamin D deficient.

After 24 months, the 25(OH)D levels were again determined, and these levels were related to how many patients developed type 2 diabetes. The results showed no significant benefit of vitamin D supplementation compared to the placebo group with respect to the risk of developing type 2 diabetes (*p* = 0.12). The 25(OH)D levels in the active treatment group increased significantly from a mean of 27.7 ng/mL to 54.3 ng/mL, while the levels in the placebo group did not change significantly with 28.2 ng/mL at the start and 28.8 ng/mL at the end of the study, respectively.

After a median follow-up of 2.5 years, the number of cases of type 2 diabetes was only slightly less with vitamin D_3_ supplementation: 293 in the vitamin D supplemented group and 323 in the placebo group, i.e., 9.39 versus 10.66 cases per 100 person years. The hazard ratio (HR) in the vitamin D group was 0.88 (95% confidence interval, CI, 0.75–1.04; *p* = 0.12). The difference was therefore not statistically significant. In a post hoc analysis of 103 patients with a baseline 25(OH)D of <12 ng/mL, the HR was 0.38 (0.18; 0.80) in the vitamin D group. In contrast, the HR was 0.92 (0.78; 1.08) in the 2319 subjects with initially higher 25(OH)D levels. According to the authors, it remained unclear from this study whether persons with a 25(OH)D level of <12 ng/mL would benefit from vitamin supplementation with regard to the risk of developing diabetes [8].

The study addressed an important question regarding diabetes prevention. Although the study by Pittas et al. [8], did not show a clear preventive effect of vitamin D with respect to the risk of diabetes in adults with evidence of pretype 2 diabetes, it must be remembered that more than 70% of the participants in this study, in both the vitamin D and the placebo groups, did not have a vitamin D deficiency. This means a substantial percent of subjects in the vitamin D group were given 4000 IUs of vitamin D daily, even though they had a normal vitamin D status. This would have diluted any potential benefit of the vitamin D, reducing the risk of progressing to type 2 diabetes since these individuals were vitamin D sufficient. The fact that they did observe a reduced risk in patients who had a 25(OH)D <12 ng/mL suggested that preventing vitamin D deficiency can reduce the risk for developing type 2 diabetes, especially in adults who have evidence for pretype 2 diabetes

To help to answer their question, in a recent randomized double-blind placebo-controlled study by Lemieux et al., 96 subjects with prediabetes, most of whom were vitamin D deficient, were given daily doses of 5000 IU vitamin D_3_ or placebo for 6 months. At the start of the study, the mean 25(OH)D level was 20.4 ng/mL (51.1 nmol/L). After 6 months, the 25(OH)D levels in the active treatment group had risen significantly, from 20.4 ng/mL to 51 ng/mL (127.6 nmol/L) compared to the placebo group (*p* < 0.001). In contrast to the placebo group, the vitamin D supplemented patients showed a significant beneficial effect on beta cell function and insulin sensitivity after 6 months (*p* = 0.009). According to the investigators of the study, vitamin D supplementation in persons with prediabetes and an inadequate vitamin D status or a vitamin D deficiency can improve insulin sensitivity and slow metabolic deterioration [7].

## 3. Vitamin D and Type 2 Diabetes

The pathogenesis of type 2 diabetes involves not only beta cell dysfunction but also insulin resistance. Various studies have indicated an association between vitamin D deficiency and the development of type 2 diabetes and the metabolic syndrome, since a vitamin D deficiency (25(OH)D < 20 ng/mL) increases insulin resistance and reduces insulin secretion from beta cells in the pancreas. There is an inverse relationship between the 25(OH)D status and the prevalence of type 2 diabetes, blood glucose concentration, and insulin resistance. Previous findings have shown that 1,25(OH)2D reduces hepatic triglyceride accumulation and glucose output under insulin-resistant conditions. Suppression of the proinflammatory cytokine TNFα by 1,25(OH)2D could be another mechanism by which improvement in vitamin D status reduces the risk for type 2 diabetes. In healthy persons, there is an inverse correlation between the serum TNFα concentration and glucose oxidation and glucose elimination (Table 1) [3,5,8,10,11,12,13].

## 4. Studies Relating Baseline Vitamin D Deficiency to Improvement in Glucose Metabolism in Response to Adequate Vitamin D Supplementation

In a randomized, placebo-controlled study with insulin-resistant South Asian women, aged 23–68 years with a median 25(OH)D of <10 ng/mL at baseline, the subjects, who were given daily supplements of 4000 IU vitamin D, showed a significant improvement in insulin sensitivity and a reduction in insulin resistance compared with those who received the placebo (*p* = 0.003 and *p* = 0.02, respectively). The reduction in insulin resistance was particularly noticeable when the 25(OH)D levels rose above 32 ng/mL (80 nmol/L). Optimal concentrations of 25(OH)D for improving the insulin resistance were between 32 ng/mL and 47.6 ng/mL (80–119 nmol/L) [12]. The results of this study showed that the benefits of vitamin D supplementation for improving insulin resistance depended on the baseline 25(OH)D status. Patients with a marked vitamin D deficiency (25(OH)D < 12 ng/mL) seemed to have particularly benefited from the vitamin D supplementation that was also demonstrated by Pittas et al. [8]. The target levels for 25(OH)D in both healthy persons and patients with metabolic syndrome and type 2 diabetes should be in the preferred range of 40–60 ng/mL (100–150 nmol/L) as recommended by the Endocrine Society [9,10,11,14,15].

The results of studies from Australia and Sweden substantiate the evidence for an association between vitamin D deficiency and the risk of metabolic syndrome or the progression from prediabetes to manifest type 2 diabetes.

A prospective study addressed the association between the 25(OH)D levels and the incidence of metabolic syndrome in 4164 Australian adults (aged ± 50 years). In addition to measuring the waist circumference of the study participants, data were also gathered on the classical risk factors of metabolic syndrome. After 5 years of follow-up, the research team observed a significantly higher probability of metabolic syndrome occurring in participants with 25(OH)D levels of <18 ng/mL and 18–23 ng/mL compared to subjects with a sufficient vitamin D status of >34 ng/mL (odds ratio 1.41 and 1.74 respectively; 95% CI). They concluded that vitamin D deficiency (25(OH)D < 20 ng/mL) and vitamin D insufficiency (25(OH)D: 21–29 ng/mL) in Australian adults were associated with a significantly increased risk of developing metabolic syndrome (*p* < 0.01), insulin resistance (*p* < 0.01), large waist circumference (*p* < 0.001), and elevated glucose and triglyceride levels (*p* < 0.01) [16].

The results of another prospective study yielded further meaningful data suggesting that vitamin D deficiency accelerated the progression of prediabetes to type 2 diabetes. In this study, the scientists investigated the glucose tolerance and 25(OH)D levels of 980 women and 1398 men, aged 35–56 years, who did not have type 2 diabetes before the start of the study. After 8–10 years of follow-up, subjects with prediabetes or type 2 diabetes were compared with controls that had normal glucose tolerance but were correlated with respect to age and sex. After adjustment for potential confounding variables, the male study participants in the highest quartile of 25(OH)D levels had a 48% lower risk of progression from prediabetes to type 2 diabetes compared with the men in the lowest quartile (odds ratio, OR 0.52, 95% CI 0.30, 0.90). Men and women who had prediabetes at the start of the study showed a remarkable 25% reduction in the incidence of type 2 diabetes for each incremental increase of 4 ng/mL (10 nmol/L) in the 25(OH)D levels. The results of this study agreed with those of a prospective study from Sweden on 24,098 women who showed a 30% reduced risk of type 2 diabetes in the group with the highest exposure to sunlight [17,18].

## 5. Conclusions

With the incidence of obesity and type 2 diabetes on the rise worldwide, it is prudent that health care professionals be aware of the association of vitamin D deficiency with increased risk for insulin resistance and type 2 diabetes. Vitamin D deficiency and insufficiency is highly prevalent worldwide. Although there is a blood test for 25(OH)D which will determine a person’s vitamin D status, it can be costly and may not be easily available. To be cost-effective, it may be reasonable to recommend appropriate vitamin D supplementation along the guidelines recommended by the Endocrine Society. They recommend daily doses of 400–1000 IUs, 600–1000 IUs, and 1500–2000 IUs of vitamin D to maintain blood levels of 25(OH)D >30 ng/mL. Obese adults require 2–3 times more vitamin D to maintain blood levels of 25(OH)D >30 ng/mL because of the uptake of vitamin D into the large body fat stores [9,19]. Numerous studies have shown that providing this amount of vitamin D to healthy adults who are vitamin D sufficient does not increase risk for toxicity [9,15], as was also demonstrated in the study by Pittas et al. [8]. The preponderance of the literature does strongly support the need to improve the vitamin D status of children and adults worldwide, including those who are at increased risk for developing type 2 diabetes.

## Figures and Tables

**Table 1:** Effects of 1,25(OH)_2_D on insulin and glucose metabolism (in vivo, in vitro).

Pancreas	Production, release, and utilization of insulin in the cells ↑
Insulin	Insulin sensitivity of the cells ↑, glucose tolerance ↑, insulin resistance ↓
AGEs	Protein glycosylation ↓, formation of advanced glycation end products (AGEs) that damage vessels and nerves ↓
Blood lipids	Cholesterol and triglyceride levels ↓, low-density lipoprotein; LDL (low density lipoprotein) oxidation ↓, effectiveness of cholesterol-lowering drugs ↑
Inflammation	Formation of proinflammatory substances such as TNFα ↓
Immune regulation	Th17/Th1 ↓, Th2/T_reg_ ↑
Vessels and nerves	Tendency to inflammation in blood vessels ↓, lipid deposition in the vessel walls ↓, vascular protective agents (e.g., IL-10) ↑
Blood pressure	Negative endocrine regulator of the RAS (rennin angiotensin system), blood pressure ↓, elasticity of the vessel walls ↑
